# Microparticles from β-thalassaemia/HbE patients induce endothelial cell dysfunction

**DOI:** 10.1038/s41598-018-31386-6

**Published:** 2018-08-29

**Authors:** Wasinee Kheansaard, Kunwadee Phongpao, Kittiphong Paiboonsukwong, Kovit Pattanapanyasat, Pornthip Chaichompoo, Saovaros Svasti

**Affiliations:** 1 Thalassemia Research Center, Institute of Molecular Biosciences, Mahidol University, Nakhon Pathom, Thailand; 2 Molecular Medicine Graduate Program, Faculty of Science, Mahidol University, Bangkok, Thailand; 3 Siriraj Center of Research Excellence for Microparticle and Exosome in Diseases, Faculty of Medicine Siriraj Hospital, Mahidol University, Bangkok, Thailand; 4 Department of Pathobiology, Faculty of Science, Mahidol University, Bangkok, Thailand; 5 Department of Biochemistry, Faculty of Science, Mahidol University, Bangkok, Thailand

## Abstract

Thromboembolic complication occurs frequently in β-thalassaemia/HbE patients, particularly in splenectomised patients. Endothelial cells play an important role in thrombosis. There is strong evidence of endothelial cell activation and dysfunction in β-thalassaemia. Microparticles (MPs) are associated with thrombosis and endothelial cell dysfunction in many diseases including β-thalassaemia. However, the effect of thalassaemic-MPs on endothelial cells mediating thrombus formation has not been elucidated. In this study, the effects of circulating MPs from β-thalassaemia/HbE patients on endothelial cell functions were investigated. The results showed that MPs directly induce tissue factor, interleukin (IL)-6, IL-8, intracellular adhesion molecule-1, vascular cell adhesion molecule-1 and E-selectin expression in human umbilical vein endothelial cells (HUVECs). Notably, the levels of these endothelial cell activation markers were significantly increased in HUVECs treated with MPs obtained from splenectomised β-thalassaemia/HbE patients when compared to MPs from non-splenectomised patients or normal subjects. The increased endothelial cell activation ultimately lead to increased monocyte-endothelial cell adhesion. THP-1 and HUVECs adhesion induced by MPs from normal subjects, non-splenectomised and splenectomised patients increased to 2.0 ± 0.4, 2.3 ± 0.4 and 3.8 ± 0.4 fold, respectively when compared to untreated cells. This finding suggests that MPs play an important role on thrombosis and vascular dysfunction in β-thalassaemia/HbE disease, especially in splenectomised cases.

## Introduction

Thromboembolic events are one of the major complications leading to significant morbidity and mortality in β-thalassaemia. The patients have an increased risk of cerebral thrombosis, deep venous thrombosis, pulmonary embolism and recurrent arterial occlusion^1^. An epidemiology study of thromboembolic events in 8,860 β-thalassaemia patients, the largest clinical study to date, demonstrated that thromboembolic events occurred in 0.9% of β-thalassaemia major (β-TM) and 3.9% of β-thalassaemia intermedia (β-TI) patients^2^. Other studies showed thromboembolic events in 0.9–3.3% of β-TM and 3.9–29% in β-TI, the frequency in β-TI was 4 to 7 times higher than β-TM^2–5^. Furthermore, the average haemoglobin (Hb) level in 67.5% of β-TI patients who had thromboembolic events was lower than 9.0 g/dL. Among these patients, 33.3% were receiving regular red blood cell (RBC) transfusions, and 94% were splenectomised^2^. The incidence of thromboembolic events might be even higher as the prevalence of silent ischemic lesions in β-thalassaemia patients, especially in splenectomised adults, was about 60% of both β-TI and β-TM^6,7^. The molecular and cellular mechanisms contributing to hypercoagulable states in thalassaemia are diverse, including abnormal RBCs, chronic platelet activation, dysregulation of haemostasis and endothelial cell dysfunction^1^.

The endothelium is a key regulator of vascular homeostasis. Activated endothelial cells tend to prothrombotic properties through dysregulation of expression of anticoagulant and procoagulant factors, adhesion molecules and proinflammatory cytokines and chemokines. There is strong evidence of endothelial cell activation and impaired function in β-thalassaemia patients. Significantly increased of activation circulating endothelial cells and soluble endothelial dysregulation/injury markers, such as tumour necrosis factor-α (TNF-α), IL-6, IL-1β, intracellular adhesion molecule-1 (ICAM-1), vascular cell adhesion molecule-1 (VCAM-1), E-selectin and von Willebrand factor (vWF) have been observed in β-thalassaemia patients^8,9^.

An elevated number of MPs in circulation is associated with thrombosis and vascular dysfunction in various diseases including diabetes mellitus, pulmonary hypertension and sickle cell disease^10–12^. In β-thalassaemia, changes in the RBC membrane and chronic platelet activation lead to the release of MPs. Moreover, the levels of MPs in splenectomised β-thalassaemia/HbE patients have been reported to be higher than those of non-splenectomised patients and normal individuals^13,14^. Interestingly, the level of MPs was correlated with platelet factor 3-like activity and prothrombinase complex activity^14^. We have previously shown that MPs obtained from β-thalassaemia/HbE patients induced platelet activation, platelet aggregation and platelet-neutrophil aggregation^15^. MPs are capable of interacting with other cells in the circulatory system that participate in various physiologic and pathologic processes, including the endothelial cells that line the inner surface of the vasculature. Herein, we have reported that β-thalassaemia/HbE-MPs enhanced endothelial dysfunction by increasing the expression of coagulation protein, pro-inflammatory cytokines and adhesion molecules ultimately leading to the increased adhesion of monocytes to endothelial cells.

## Results

### Haematological and microparticle profiles in blood circulation of β-thalassaemia/HbE patients

Haematological analysis revealed that β-thalassaemia/HbE patients had an anaemia phenotype with increased numbers of platelets and MPs (annexin V+ MPs) as described in previous studies^13–15^. The number of MPs originating from platelets and RBCs of β-thalassaemia/HbE was higher than normal-MPs. Interestingly, endothelial cell-derived MPs (ECMPs), a hallmark of endothelial dysfunction, are also increased significantly in β-thalassaemia/HbE patients both non-splenectomised (2.9 ± 0.9 × 10^3^ particles/µL) and splenectomised (9.5 ± 3.0 × 10^3^ particles/µL) when compared to normal subjects (0.3 ± 0.7 × 10^3^ particles/µL) (*P* < 0.05) (Table 1). The increased circulating MPs in β-thalassaemia/HbE patients could disturb endothelial cell functions including the induction of coagulation molecules, cytokines and adhesion molecule expression.

**Table 1:** Haematological parameters and microparticle profiles.

Description	Normal subjects			β-Thalassaemia/HbE patients					
				Non-splenectomy			Splenectomy		
Number	16			21			11		
Red blood cell count (×10^6^/μL)	4.7	±	0.4	3.9	±	1.0^a^	3.2	±	0.5^a^
Haemoglobin (g/dL)	12.7	±	1.4	7.2	±	1.5^a^	7.1	±	1.0^a^
Haematocrit (%)	38.6	±	3.0	24.3	±	4.5^a^	24.2	±	2.7^a^
MCV (fL)	82.6	±	6.6	62.8	±	5.5^a^	76.0	±	7.5
MCH (pg)	27.3	±	2.9	18.6	±	2.2^a^	22.0	±	3.2^a^
MCHC (g/dL)	32.9	±	1.4	29.5	±	1.9^a^	28.9	±	1.9^a^
Red cell distribution width (%)	13.0	±	1.8	23.9	±	2.1^a^	23.2	±	2.2^a^
Reticulocytes (%)	1.02	±	0.2	3.7	±	1.0^a^	14.0	±	6.3^a,b^
NRBCs (cells/100 WBCs)	not detectable			3.6	±	2.6^a^	326.5	±	237.2^a,b^
WBC count (×10^3^/μL)	6.7	±	2.0	7.1	±	2.1	16.9	±	4.7^a,b^
Platelet count (×10^3^/μL)	272.5	±	66.3	169.0	±	78.0	815.0	±	114.0^a,b^
Annexin V+ microparticle profiles									
Total MPs (×10^3^ particles/μL)	16.3	±	6.5	28.6	±	9.1	79.1	±	19.8^a,b^
PMPs (×10^3^ particles/μL)	5.7	±	0.8	12.0	±	2.3^a^	51.5	±	10.1^a,b^
RBCMPs (×10^3^ particles/μL)	1.3	±	0.3	4.3	±	0.9^a^	11.1	±	2.1^a,b^
WBCMPs (×10^3^ particles/μL)	0.7	±	0.2	1.4	±	0.3	5.5	±	1.6^a,b^
ECMPs (×10^3^ particles/μL)	0.3	±	0.7	2.9	±	0.9^a^	9.5	±	3.0^a,b^

ECMPs; endothelial cell-derived MPs, MCV; mean corpuscular volume, MCH; mean corpuscular haemoglobin, MCHC; mean corpuscular haemoglobin concentration, MPs; microparticles, NRBCs; nucleated red blood cells, PMPs; platelet-derived MPs, RBCMPs; RBC-derived MPs, WBCs; white blood cells and WBCMPs; WBC-derived MPs. ^a^Significant difference when compared to normal subjects at *P* < 0.05. ^b^Significant difference when compared to non-splenectomised β-thalassaemia/HbE patients at *P* < 0.05.

### Microparticles are internalised by HUVECs

**Figure 1:**
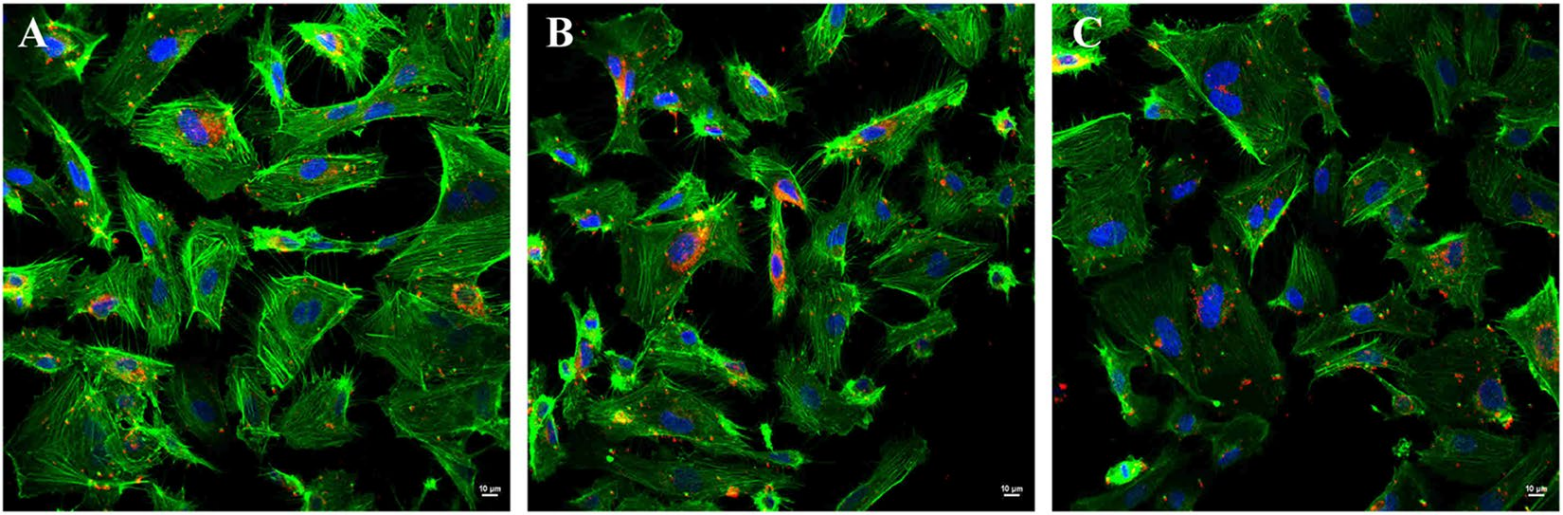
MPs are internalised by HUVECs. HUVECs were incubated with PKH26 labeled MPs for 2 h at 37 °C. Representative confocal microscopy images of HUVECs cytoplasm (green) and nuclei (blue) on incubation with MPs (red) obtained from normal subjects (**A**), non-splenectomised β-thalassaemia/HbE (**B**) and splenectomised β-thalassaemia/HbE patients (**C**).

The ability of HUVECs to internalise MPs was examined. Confocal microscopy imaging revealed well defined red dots, corresponding to PKH26-labeled MPs (red fluorescence), in the cytoplasm of HUVECs after exposure to the PKH26-labeled MPs for 2 h. The amount of internalised MPs was not different amongst MPs obtained from normal subjects, non-splenectomised and splenectomised β-thalassaemia/HbE patients (Fig. 1).

### Microparticles induced coagulation protein expression in HUVECs

**Figure 2:**
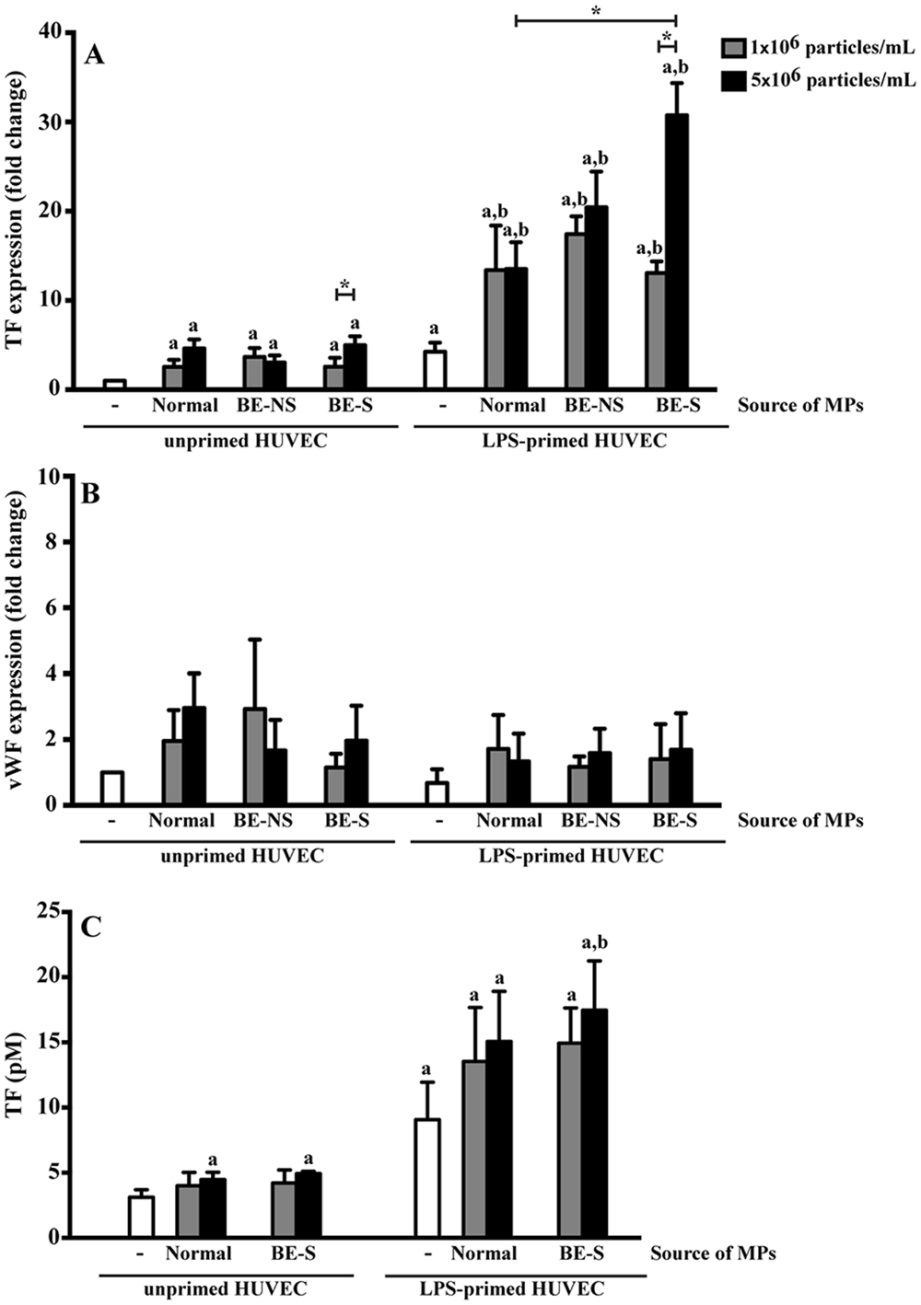
MPs induced coagulation protein expression. Unprimed and LPS-primed HUVECs were treated with 1 × 10^6^ and 5 × 10^6^ particles/mL MPs obtained from 7 normal subjects, 9 non-splenectomised β-thalassaemia/HbE patients (BE-NS) and 9 splenectomised β-thalassaemia/HbE patients (BE-S). The expression of (**A**) tissue factor (TF) and (**B**) von Willebrand factor (vWF) were determined by RT-qPCR. (**C**) TF activity in supernatant secreted by HUVECs. Unprimed and LPS-primed HUVECs were treated with 1 × 10^6^ and 5 × 10^6^ particles/mL MPs obtained from 3 normal subjects and 3 splenectomised β-thalassaemia/HbE patients (BE-S). ^a^Statistically significant difference when compared with unprimed HUVECs at *P* < 0.05. ^b^Statistically significant difference when compared with LPS-primed HUVECs at *P* < 0.05. *Statistically significant difference between groups at *P* < 0.05. Each experiment was duplicated.

The coagulation cascade is a haemostatic process that serves to limit the amount of blood loss when wounding occurs. However, derangements of this process can result in thrombosis. To determine the ability of MPs to induce coagulation protein expression, HUVECs were cultured in the presence of MPs at a concentration of 1 × 10^6^ or 5 × 10^6^ particles/mL. At about three folds less than the physiological number of MPs in circulation of the normal subjects (5 × 10^6^ particles/mL), the MPs from both non-splenectomised (3.0 ± 0.8 folds) and splenectomised (4.9 ± 1.0 folds) β-thalassaemia/HbE patients and normal subjects (4.6 ± 0.9 folds) significantly increased tissue factor (TF) expression in HUVECs compared to the untreated baseline (*P* < 0.05) (Fig. 2A). By contrast, these MPs had no effect on vWF expression (Fig. 2B). HUVECs treated with lipopolysaccharide (LPS), a common activator promoting an inflammatory response of endothelial cells, also resulted in significantly increased TF expression (4.3 ± 1.1 folds) (*P* < 0.05) but not vWF (Fig. 2). Interestingly, HUVECs in combination with LPS and MPs led to an increase in TF expression as compared to HUVECs treated with either LPS or MPs alone. In particular, MPs obtained from splenectomised patients could induce the highest expression of TF. At 5 × 10^6^ particles/mL, TF expression induced by MPs from splenectomised patients (30.3 ± 3.6 folds) was significantly higher than those induced by MPs from non-splenectomised patients (20.2 ± 4.0 folds) and normal subjects (13.6 ± 2.7 folds) (*P* < 0.05) (Fig. 2A).

TF activity in the supernatant secreted by HUVECs was assessed by the ability to convert coagulation factor X (FX) to activated FX (FXa) in the presence of FVIIa. Increased TF activity was observed in HUVECs treated with 5 × 10^6^ particles/mL MPs from both normal subjects (4.5 ± 0.6 pM) and splenectomised β-thalassaemia/HbE patients (4.9 ± 0.2 pM) when compared to the untreated baseline (3.1 ± 0.6 pM) (*P* < 0.05) in line with mRNA levels (Fig. 2C). HUVECs treated with a combination of LPS and MPs from splenectomised patients (17.5 ± 3.8 pM) and normal subjects (15.1 ± 3.8 pM) led to an increase in TF activity compared to HUVECs treated with LPS (9.1 ± 2.9 pM) or MPs alone (*P* < 0.05). While there was no statistical significance, there was a trend of increased TF activity in HUVECs treated with MPs from splenectomised patients as compared with normal subjects (Fig. 2C).

### Microparticles induced pro-inflammatory cytokine expression in HUVECs

Endothelial cells release cytokines and chemokines such as TNF-α, IL-1β, IL-6 and IL-8 to mediate inflammation and the coagulation response when cells are injured. Overproduction of these cytokines by endothelial cells results in chronic inflammation and thrombus formation. The effects of MPs on cytokine production by HUVECs were thus measured using RT-qPCR and ELISA.

**Figure 3:**
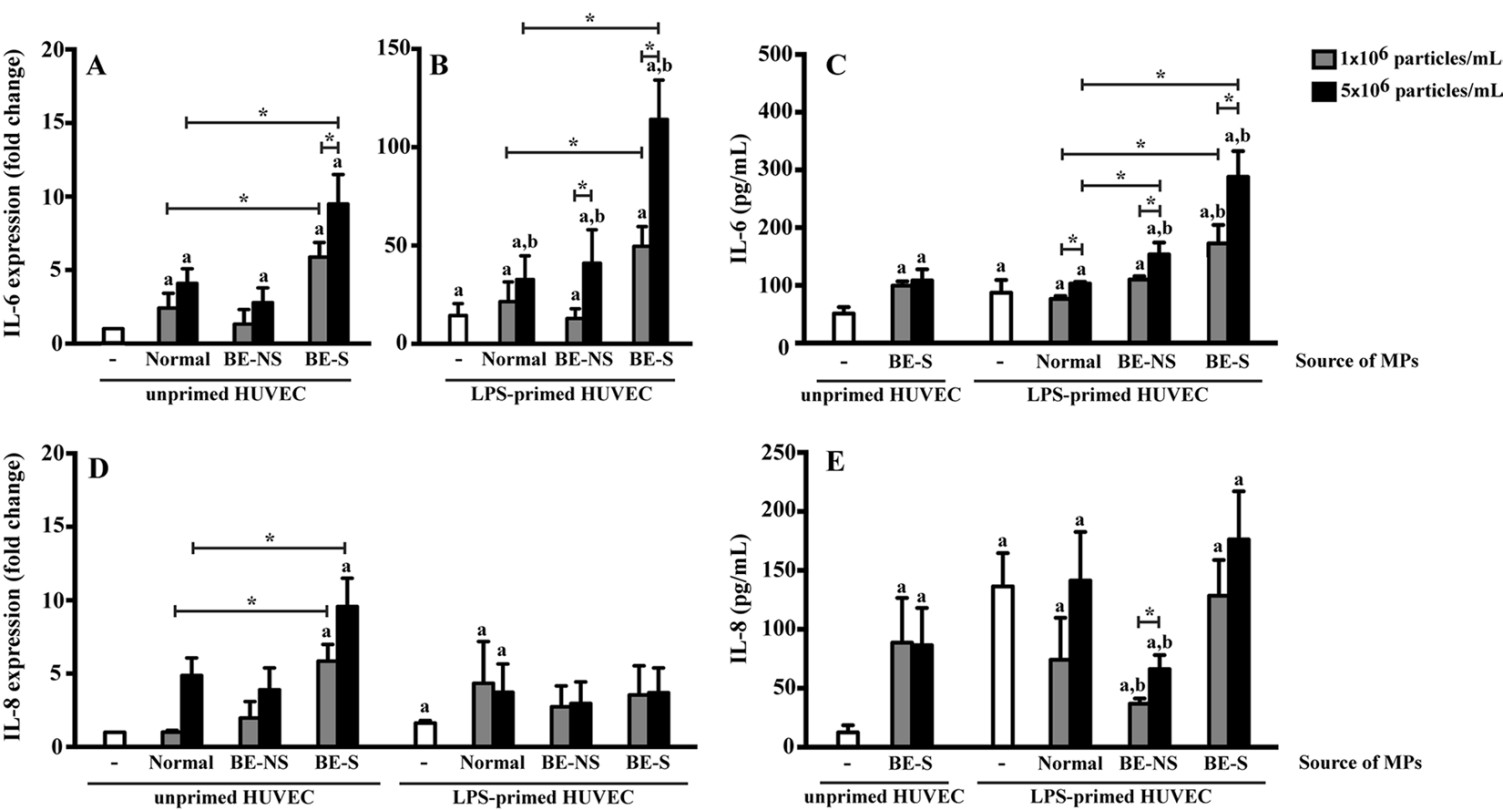
MPs induced pro-inflammatory cytokine expression. Unprimed and LPS-primed HUVECs were treated with 1 × 10^6^ and 5 × 10^6^ particles/mL MPs obtained from 7 normal subjects, 9 non-splenectomised β-thalassaemia/HbE patients (BE-NS) and 9 splenectomised β-thalassaemia/HbE patients (BE-S). IL-6 and IL-8 production measured as the mRNA levels (**A**,**B**,**D**, respectively) and protein levels (**C**,**E**, respectively) in HUVECs and supernatant were analysed by RT-qPCR and ELISA, respectively. ^a^Statistically significant difference when compared with unprimed HUVECs at *P* < 0.05. ^b^Statistically significant difference when compared with LPS-primed HUVECs at *P* < 0.05. *Statistically significant difference between groups at *P* < 0.05. Each experiment was duplicated.

LPS primed HUVECs, as a positive control, showed significantly increased IL-6 and IL-8, at both the mRNA and protein levels, when compared to the untreated HUVECs (Fig. 3). HUVECs treated with MPs resulted in increased IL-6 and IL-8 mRNA levels when compared to the untreated HUVECs (Fig. 3A,D). At 5 × 10^6^ particles/mL, IL-6 mRNA level in HUVECs treated with MPs from normal subjects, non-splenectomised patients and splenectomised patients (4.1 ± 1.0, 2.8 ± 1.1 and 9.5 ± 2.0 folds, respectively) was significantly increased, when compared to the untreated HUVECs (*P* < 0.05) (Fig. 3A). The IL-6 mRNA level observed in HUVECs treated with combined LPS and MPs was higher than HUVECs treated with MPs alone. At 5 × 10^6^ particles/mL, IL-6 mRNA level in HUVECs treated with combined LPS and MPs from normal subjects, non-splenectomised patients and splenectomised patients was significantly increased, 32.7 ± 12.0, 41.0 ± 17.1 and 114.1 ± 20.1 folds, respectively compared to HUVECs treated with LPS (14.4 ± 6.2 folds) or MPs alone (*P* < 0.05) (Fig. 3B).

ELISA analysis for IL-6 and IL-8 levels demonstrated that MPs induced both IL-6 and IL-8 secretion from HUVECs (Fig. 3C,E). Interestingly, at 5 × 10^6^ particles/mL, IL-6 expression induced by MPs from splenectomised patients (288.1 ± 44.5 pg/mL) was significantly higher than those induced by MPs from non-splenectomised patients (153.8 ± 20.4 pg/mL) and normal subjects (103.1 ± 2.9 pg/mL) (*P* < 0.05) (Fig. 3C). In general, IL-6 responds mainly to acute inflammation and acts as a growth factor to promote cell-mediated immune responses, while IL-8 responds mainly to leukocyte recruitment to the site of inflammation. These data indicate that splenectomised-MPs have the ability to induce pro-inflammatory cytokine production by HUVECs.

### Microparticles induced adhesion molecule expression in HUVECs

The cell-to-cell interaction between leukocytes and endothelial cells that occurs during inflammation is mediated by adhesion molecules. To initiate the leukocyte rolling and migration from the blood circulation to the site of inflammation, activated endothelial cells expose adhesion molecules such as VCAM-1, E-selectin and ICAM-1 on their surface.

**Figure 4:**
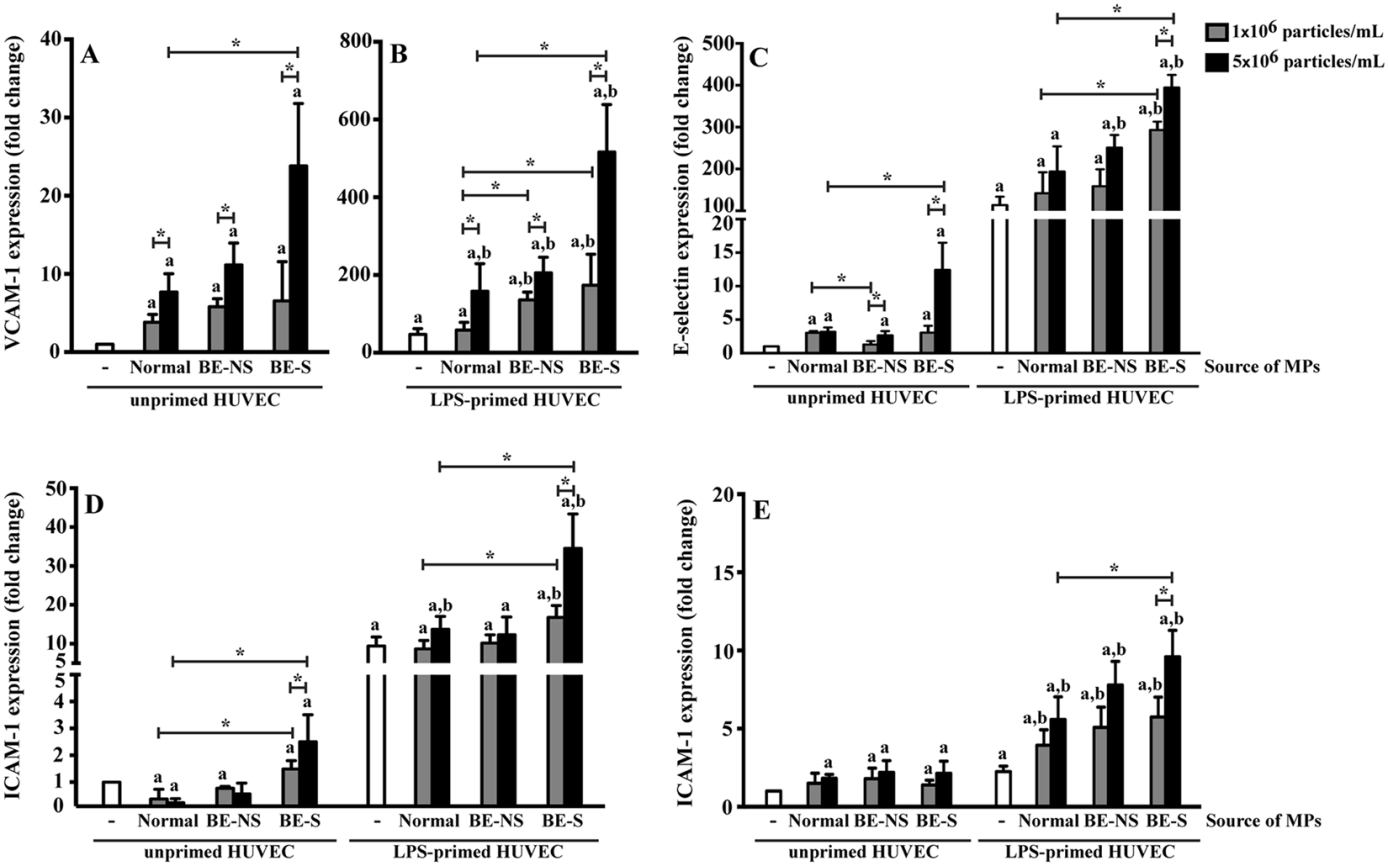
MPs induced adhesion molecule expression. Unprimed and LPS-primed HUVECs were treated with 1 × 10^6^ and 5 × 10^6^ particles/mL MPs obtained from 7 normal subjects, 9 non-splenectomised β-thalassaemia/HbE patients (BE-NS) and 9 splenectomised β-thalassaemia/HbE patients (BE-S). HUVECs were analysed for the expression of (**A**,**B**) VCAM-1 mRNA, (**C**) E-selectin mRNA and (**D**) ICAM-1 mRNA using RT-qPCR. (**E**) Flow cytometric analysis of ICAM-1 expression on HUVECs were also performed. ^a^Statistically significant difference when compared with unprimed HUVECs at *P* < 0.05. ^b^Statistically significant difference when compared with LPS-primed HUVECs at *P* < 0.05. *Statistically significant difference between groups at *P* < 0.05. Each experiment was duplicated.

MPs treatment caused a significant increase in VCAM-1, E-selectin and ICAM-1 mRNAs in HUVECs (Fig. 4A,C,D). At 5 × 10^6^ particles/mL unprimed HUVECs treated with MPs from normal subjects, non-splenectomised and splenectomised patients resulted in increased expression of VCAM-1 (7.7 ± 2.4, 11.1 ± 2.8 and 23.8 ± 8.0 folds) and E-selectin (3.2 ± 0.7, 2.6 ± 0.6 and 12.4 ± 4.1 folds) when compared with that of unprimed HUVECs (*P* < 0.05). While unprimed HUVECs treated with 5 × 10^6^ particles/mL MPs from splenectomised patients resulted in increased expression of ICAM-1 (2.5 ± 1.0 folds) when compared with that of unprimed HUVECs (*P* < 0.05). LPS primed HUVECs also resulted in the significantly increased VCAM-1, E-selectin and ICAM-1 mRNAs (47.1 ± 15.1, 113.5 ± 20.1 and 9.4 ± 2.3 folds) when compared with those of unprimed HUVECs (*P* < 0.05) (Fig. 4B–D). In addition, the combined LPS and MPs significantly induced the adhesion molecule expression of HUVECs in a dose dependent manner with a significantly higher level when compared to HUVECs treated with LPS or MPs alone. At 5 × 10^6^ particles/mL MPs from normal subjects, non-splenectomised and splenectomised patients increase VCAM-1 mRNA expression of LPS-primed HUVECs (157.6 ± 71.1, 205.0 ± 40.2, and 515.9 ± 122.1 folds) when compared to primed HUVECs with LPS alone (*P* < 0.05) (Fig. 4B). Combined LPS and 5 × 10^6^ particles/mL MPs from splenectomised patients was resulting in the increasing of E-selectin (394.5 ± 30.2 folds) and ICAM-1 (34.5 ± 8.9 folds) mRNA expression when compared to primed HUVECs with LPS alone (*P* < 0.05) (Fig. 4C,D).

Flow cytometric analysis of ICAM-1 expression showed a result consistent with the RT-qPCR analysis suggesting that MPs significantly stimulated ICAM-1 expression as compared to the untreated cells (Fig. 4E). The results also demonstrated that the combined MP and LPS treated HUVECs significantly increased ICAM-1 expression when compared to untreated HUVECs and HUVECs treated with LPS alone (Fig. 4E). It is noteworthy that the effect of MPs (at 5 × 10^6^ particles/mL) obtained from splenectomised patients (9.6 ± 1.7 folds) was significantly higher than MPs obtained from normal subjects (5.6 ± 1.5 folds) in LPS-primed HUVECs (*P* < 0.05). While the effects of non-splenectomised-MPs on HUVECs were not significantly different from normal-MPs.

### Microparticles stimulate monocyte-endothelial cell adhesion

The final step of the coagulation and inflammation response on endothelial dysfunction is the initiation of thrombus formation via the leukocyte-endothelial cell aggregation. As thalassaemia MPs could induce TF, IL-6, IL-8, ICAM-1, VCAM-1 and E-selectin expression, the potential functional consequences of MPs influence on the adhesion of endothelial cells and monocytes was examined.

**Figure 5:**
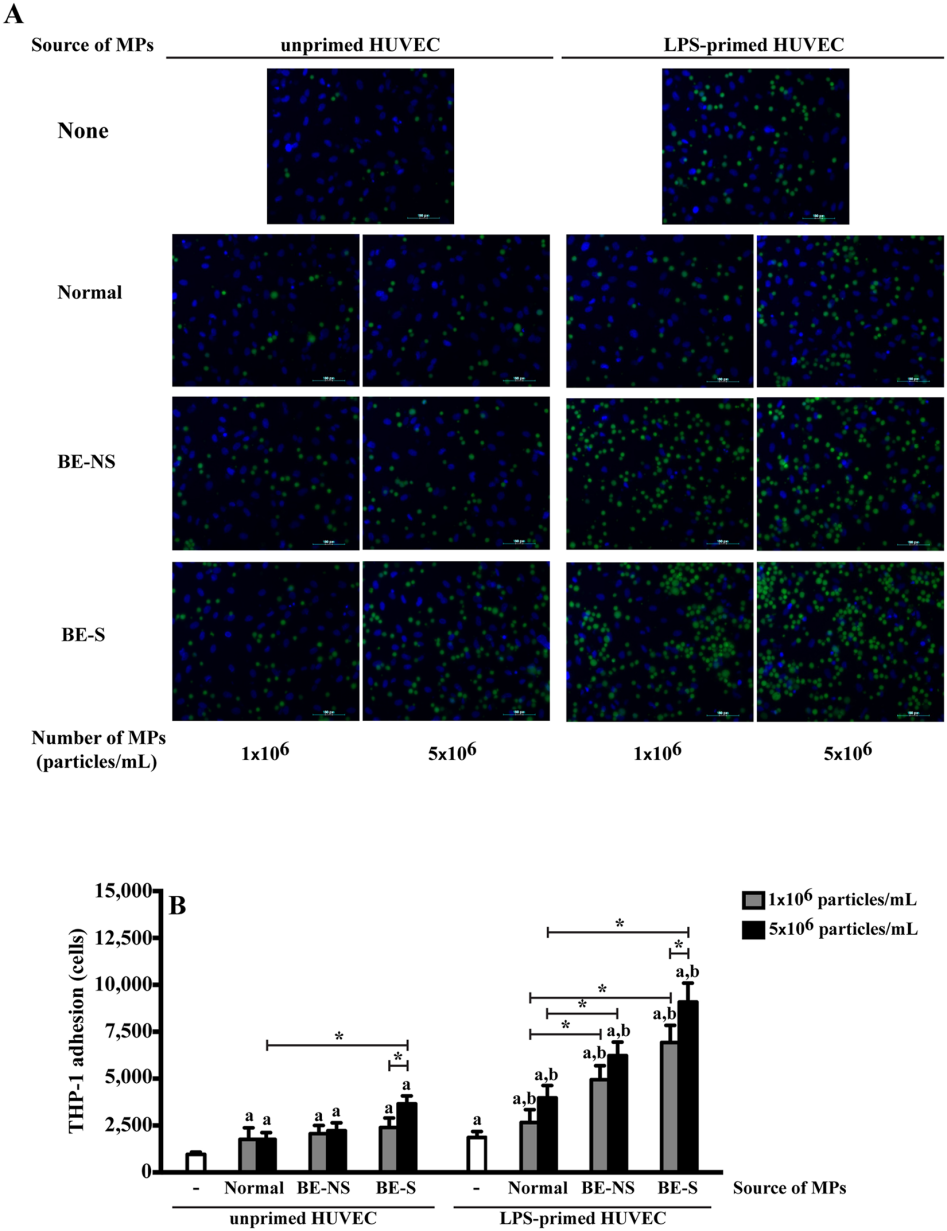
MPs stimulate monocyte-endothelial cell adhesion. Unprimed and LPS-primed HUVECs were treated with 1 × 10^6^ and 5 × 10^6^ particles/mL MPs obtained from 7 normal subjects, 9 non-splenectomised β-thalassaemia/HbE patients (BE-NS) and 9 splenectomised β-thalassaemia/HbE patients (BE-S). HUVECs were stained with Hoechst (blue), and BCECF-AM-labeled THP-1 cell lines (green). (**A**) The illustration was captured fluorograph at ×200 magnification by fluorescent microscopy. (**B**) The fluorescent intensity of the amount of adhered THP-1 cells was detected by fluorescent plate reader and calculated by normalised with untreated HUVECs. ^a^Statistically significant difference when compared with unprimed HUVECs at *P* < 0.05. ^b^Statistically significant difference when compared with LPS-primed HUVECs at *P* < 0.05. *Statistically significant difference between groups at *P* < 0.05. Each experiment was duplicated.

The splenectomised-MPs induced human monocytic leukemia cell line (THP-1) cell adhesion to HUVECs in a dose-dependent manner was clearly evident by fluorescent microscopy in cells both unprimed and primed with LPS (Fig. 5A). In unprimed HUVECs, 5 × 10^6^ particles/mL MPs from normal subjects, non-splenectomised and splenectomised patients had the ability to induce THP-1 and HUVECs adhesion at 1,950 ± 622, 2,222 ± 422 and 3,656 ± 421 cells, respectively (Fig. 5B). This translated to 2.0 ± 0.4, 2.3 ± 0.4 and 3.8 ± 0.4 folds increases when compared to the untreated cells, respectively. While HUVECs primed with LPS increased THP-1 and HUVECs adhesion when compared to unprimed cells. THP-1 adherence to primed HUVECs with 5 × 10^6^ particles/mL MPs from normal subjects, non-splenectomised and splenectomised patients were 3,973 ± 661, 6,226 ± 722 and 9,090 ± 1,004 cells, respectively (Fig. 5B). This was calculated to 2.1 ± 0.6, 3.4 ± 0.6 and 4.9 ± 0.7 fold increases when compared to LPS primed HUVECs, respectively. It is noteworthy that MPs obtained from splenectomised patients significantly induced THP-1 and HUVECs adhesion at a higher level than MPs obtained from normal subjects and non-splenectomised patients, both unprimed and primed with LPS.

### Effect of phosphatidylserine (PS) on microparticles to endothelial cell activation

To address whether PS exposed on MPs outer surface might be a factor that induces the coagulation and inflammation response of HUVECs, MPs were pre-incubated with the PS antagonist, annexin V (5 and 10 μg/mL) to prevent PS exposure from the MP surface. The result showed that PS-bearing MPs were reduced about 94% (Fig. S1). However, there were no significant differences of TF, IL-6 and ICAM-1 mRNA levels between HUVECs treated with annexin V− MPs and control MPs in both LPS primed and unprimed HUVECs (Fig. S2). The splenectomised-MPs treated with annexin V still had the highest effect on stimulation of TF, IL-6 and ICAM-1 expression (25.7 ± 11.4, 90.2 ± 18.2 and 27.7 ± 9.8 folds, respectively) in LPS primed HUVECs when compared to MPs obtained from normal individuals (13.3 ± 5.1, 28.7 ± 3.1 and 13.9 ± 4.7 folds, respectively) (*P* < 0.05). This suggested that the intrinsic properties in splenectomised MPs, other than PS exposure, may differ from those of non-splenectomised-MPs and normal-MPs, and be able to activate endothelial cells.

### Correlation between microparticle origin and endothelial cell activation markers

Since MPs in circulation may derive from RBCs or platelets, the effect of MPs origin on endothelial cell activation markers was examined. The Spearman’s correlation coefficient of various MPs origins against mRNA levels of endothelial cell activation markers are shown in Table 2 and Fig. S3. Platelet-derived MPs (PMPs) were significantly correlated with adhesion molecule expression in both unprimed and LPS-primed HUVECs. While, RBC-derived MPs (RBCMPs) and ECMPs were not significantly correlated with adhesion molecules expression. This suggested that PMPs were the key inducer of adhesion molecules expression. Interestingly, PMPs were also significantly correlated with TF and IL-6 expression in LPS-primed HUVECs. ECMPs were only significantly correlated with TF expression in LPS-primed HUVECs.

**Table 2:** Correlation between MPs origin and endothelial cell activation markers.

	PMPs	RBCMPs	ECMPs
Unprimed HUVECs			
ICAM-1	r_s_ = 0.904, *P* < 0.001	n.s.	n.s.
VCAM-1	r_s_ = 0.922, *P* < 0.001	n.s.	n.s.
E-selectin	r_s_ = 0.821, *P* < 0.001	n.s.	n.s.
Tissue factor	n.s.	n.s.	n.s.
IL-6	n.s.	n.s.	n.s.
IL-8	n.s.	n.s.	n.s.
LPS-primed HUVECs			
ICAM-1	r_s_ = 0.809, *P* < 0.001	n.s.	n.s.
VCAM-1	n.s.	n.s.	n.s.
E-selectin	r_s_ = 0.911, *P* < 0.001	n.s.	n.s.
Tissue factor	r_s_ = 0.907, *P* < 0.001	n.s.	r_s_ = 0.734, *P* < 0.001
IL-6	r_s_ = 0.907, *P* < 0.001	n.s.	n.s.
IL-8	n.s.	n.s.	n.s.

ECMP; endothelial cell-derived MPs, HUVECs; human umbilical vein endothelial cells, ICAM-1; intracellular adhesion molecule-1, IL; interleukin, LPS; lipopolysaccharide, MPs; microparticles, n.s.; not significance, no correlation, PMPs; platelet-derived MPs, RBCMPs; red blood cell-derived MPs, r_s_; Spearman’s coefficient correlation and VCAM-1; vascular cell adhesion molecule-1.

## Discussion

Thromboembolic events are a typical complication and major cause of death in β-thalassaemia patients, particularly in splenectomised β-TI^1,2^ and β-thalassaemia/HbE^16^. The pathogenesis of the hypercoagulable state in β-thalassaemia occurs from degradation of excess α-globin chains in RBCs resulting in the accumulation of intracellular labile iron, and leading to oxidative stress and more rigid and deformed RBCs, with consequent premature destruction. This process is associated with loss of the normal asymmetrical distribution of the membrane phospholipids and exposure of PS on the outer surface of the RBC membrane, resulting in formation of tenase and prothrombinase complexes and thrombin generation. The enhanced thrombin generation leads to the activation of platelets, monocytes, granulocytes and endothelial cells which further contribute to thrombus formation^1,17^. Despite the appearance of these anomalies, the mechanism of the hypercoagulable state in β-thalassaemia is incompletely understood. MPs derived from RBCs and platelets have been reported to play an important role in homeostasis involving coagulation, inflammation and vascular function. Therefore, MPs could be an important jigsaw piece in the mechanism of the hypercoagulable state in β-thalassaemia.

MPs are involved in the hypercoagulable stage through different mechanisms including interference with coagulation pathways, modulation of inflammation and inducing adherence of platelets and other cells to endothelial cells. MPs from patients with type 2 diabetes increased coagulation activity^18^ and those isolated from atherosclerotic plaques increased endothelial adhesion molecule expression and monocyte recruitment^19^. Intravenous administration of sickle cell disease MPs induced vaso-occlusion, stimulated cellular adhesion to endothelial cells and favoured endothelial dysfunction in a murine model of sickle cell disease^20^. In the present study, we have shown that MPs induced expression of coagulation proteins, pro-inflammatory cytokines and adhesion molecules, and ultimately led to an increased monocyte-endothelial cell adhesion.

In this study, the amount of MPs (5 × 10^6^ particles/mL) used was about three fold lower than the physiological number of MPs in circulation in normal subjects (1.6 × 10^7^ particles/mL). However, the percentages of platelet activation in platelets from splenectomised patients treated with splenectomised-MPs at 10-fold lower to 100-fold higher than physiological levels were significantly increased when compared to platelets treated with normal-MPs, and in dose dependent manner^15^. HUVECs incubated with MPs obtained from sickle cell disease (SCD) patients at about 5-fold lower than physiological levels resulted in increased reactive oxygen species production and apoptosis when compared with that of normal controls. Intravenous injection of SAD mice, a model of SCD, with 2 × 10^4^ MPs from SAD mice triggered rapid vaso-occlusions in kidneys^20^. This suggests that the effect of MPs could be observed at a lower than circulations concentrations and reflects their effects *in vivo*.

Infection and inflammation are often linked to thrombotic events. A chronic inflammatory state is present in β-thalassaemia/HbE patients, with increased levels of pro-inflammatory cytokines such as IL-6 and inflammation markers such as C-reactive protein^8,21^. Under inflammation conditions such as LPS-primed HUVECs, MPs originating from both β-thalassaemia**/**HbE and normal subjects increased the effect of MPs on pro-inflammatory cytokine secretion, expression of adhesion molecules and also TF and further recruitment of monocyte adhesion to endothelial cells. Thus, MPs may mediate the enhanced hypercoagulability in β-thalassaemia patients.

Remarkably, the MPs from β-thalassaemia patients, especially those with splenectomy, induced more pronounced changes in endothelial cells. Splenectomy could affect MPs properties in three ways, (i) increase the amounts of MPs in circulation, (ii) led to different ratios of cellular origin of MPs and (iii) affect the different biomolecule components of MPs. Loss of the spleen’s filtering activities results in disturbance of the clearance process of many cell types from circulation. Splenectomised β-thalassaemia/HbE patients have increased levels of many cell types in peripheral blood including damaged RBCs and activated platelets, which in turn generate the increased number of circulating MPs originating from those cells. Consistent with other reports, we found that MPs originating from platelets and RBCs in splenectomised β-thalassaemia/HbE patients were significantly higher than non-splenectomised patients and normal subjects^14,22,23^. In this study, the same amount of annexin V+ MPs from these three subject groups were applied to HUVECs. The results showed that splenectomised-MPs induced more endothelial activation suggesting that the intrinsic properties of the MPs could also contribute.

The component of MPs from these three subject groups may also differ due to selective processes to package biological molecules into MPs. MPs harbour large quantities of a wide range biomolecules including proteins (cytokines or other secretion proteins, plasma membrane proteins or receptors, transporter proteins, cytoskeleton and signal proteins), lipids and nucleic acids (mRNA and microRNA). The contents and characteristics of MPs derived from the same cell lineage can vary depending on the stimulus. MPs from activated neutrophils show 10-fold enrichment of Mac-1 as compared to MPs from non-activated neutrophils^24^. MPs derived from stimulated THP-1 cells contained increased inflammatory microRNA and could induce endothelial inflammation more than MPs from non-stimulated THP-1 cells^25^. The study of MP-associated microRNAs of patients with acute and stable coronary artery disease showed that patients with stable coronary artery disease had significantly less pro-inflammatory microRNAs than patients with acute coronary syndrome^25^. The different protein components in MPs has been observed^13^. However, the exact biomolecules which make splenectomised MPs to have a higher endothelial cell activation propriety remains to be determined.

Spearman’s correlation coefficient analysis showed that the increased expression of coagulation protein, pro-inflammatory cytokines and adhesion molecules was correlated with PMPs. In β-thalassaemia major patients, PMPs but not RBCMPs have been shown to be procoagulant^26^. Indeed, PMPs certainly play additional roles in normal and pathological hemostasis, particularly in cell-to-cell interaction and generation of thrombus formation^27,28^. As shown in patients with peripheral artery disease, elevated levels of PMPs were associated with increased plasma soluble P-selectin levels, and PMPs markedly acted as a potential influence to the progression of atheroma^29^. Moreover, Merten, *et al*. proposed that PMPs, attached to subendothelium and activated endothelial cells, can recruit platelet adhesion on endothelial cells^30^. Our finding is similar to Barry, *et al*. who have reported that PMPs can activate IL-1, IL-6 and IL-8 production and ICAM-1 expression on endothelial cells and promote U-937 monocytic cell line-HUVECs interactions^31^.

We have previously shown that MPs obtained from β-thalassaemia/HbE patients induced platelet activation, platelet aggregation and platelet-neutrophil aggregation, which partly contributes to the hypercoagulable state in β-thalassaemia/HbE patients^15^. The studies presented above represent the effects of the MPs on endothelial cell activation that leads to the increased adhesion of leukocytes to endothelial cells. These findings suggest that splenectomised MPs could play an important role on endothelial cell activation/dysfunction leading to thromboembolic events in β-thalassaemia/HbE patients. As chronic platelet activation leads to the release of MPs, anti-platelets or aspirin treatment may also help to reduce PMPs in patients who had undergone splenectomy, and may help to reduce thromboembolic events.

## Methods

### Patients and blood samples

This study was performed in accordance with the Helsinki declaration and was approved by the Mahidol University Institutional Review Board (MU-IRB), approval number 2014/013.0502. Written informed consent was obtained from all individual participants included in the study. Peripheral blood samples were collected from 32 β-thalassaemia/HbE patients (21 non-splenectomy and 11 splenectomy) and 16 normal subjects at ages ranging from 23 to 45 years old. All subjects had no evidence of concurrent infection, history of vaso-occlusive episode or atherosclerotic vascular disease. Patients under treatments with aspirin, antibiotics, anti-depressants, beta-blockers and anti-platelets were excluded, and none had been hospitalised or transfused within 4 weeks. All blood samples were collected at room temperature (RT) and processed within 2–3 h.

### Isolation of microparticles from peripheral blood samples

The MPs were isolated as described in the previous study^15^. Briefly, fresh whole blood samples collected in 3.2% trisodium citrate anticoagulant were centrifuged at 1,500 × g for 15 min at 25 °C to collect platelet-poor plasma and re-centrifuged at 14,000 × g for 2 min at 4 °C to obtained platelet-free plasma (PFP). Pellet MPs were collected after centrifugation of PFP at 14,000 × g at 4 °C for another 45 min and washed once with phosphate buffer saline (PBS).

### Blocking of phosphatidylserine on microparticles surface

Isolated MPs were pre-incubated with purified annexin V (Biolegend, San Diego, CA) (final concentration at 5 and 10 µg/mL) for 15 min at 25 °C, then washed once with PBS to collect annexin V-MP pellet samples.

### Cell culture

HUVECs were cultured in endothelial cell growth medium (Cell applications, San Diego, CA) at 37 °C, 5% CO_2_. THP-1 was grown in RPMI1640 culture medium supplemented with 10% foetal bovine serum, 100 U/mL penicillin, 100 μg/mL streptomycin and 2 mM L-glutamine at 37 °C, 5% CO_2_.

### HUVECs cell treatment

HUVECs were plated in a 24-well-plate until forming 80–90% confluent monolayer and unprimed or primed with 10 μg/mL LPS (Sigma-Aldrich, St. Louis, MO) for 1 h at 37 °C, 5% CO_2_. Then, MPs (1 × 10^6^ or 5 × 10^6^ particles/mL) were added for 6 or 24 h at 37 °C, 5% CO_2_. HUVECs were washed and harvested to determine endothelial activation markers.

### Analysis of microparticle internalisation

MPs internalisation by HUVECs was examined by confocal microscopy analysis. MPs were labeled with 2 µM PKH26 red fluorescent cell linker kits (Sigma-Aldrich) for 5 min at RT. HUVECs were treated with labeled MPs at concentration 5 × 10^6^ particles/ml for 2 h at 37 °C, 5% CO_2_. Then non-adherent MPs were removed by gently washing 3 times with PBS. HUVECs were fixed with 4% formaldehyde for 30 min and stained with F-actin staining kit-green fluorescence (Abcam, Cambridge, UK) for 60 min at RT. Slides were washed and mounted using mounting buffer containing DAPI (X-ZELL, Sunnyvale, CA). Serial Z-stacked confocal imaging was performed with an A1R confocal microscope (Nikon, Tokyo, Japan), and image analysis was performed with NIS Element Viewer 4.2 software (Nikon). Single representative images of the central layers are shown.

### Flow cytometric analysis of microparticles

Total numbers and cellular origins of MPs in peripheral blood samples and pellet MP samples were quantitated using flow cytometry as described in the previous study^15^. Briefly, samples were stained with FITC or PE annexin V, FITC anti-CD41 (platelet marker), PE anti-CD105 (endothelial cell marker), PerCP anti-CD45 (leukocyte marker) and APC anti-CD235a or glycophorin A (RBC marker). All monoclonal antibodies were purchased from Becton Dickinson Biosciences (BDB, San Jose, CA). Data from at least 10,000 events were acquired and analysed with the use of FACSCalibur flow cytometer and CellQuestPro^TM^ software (BDB). MP population was determined by comparison with beads of 1 μm in diameter (Spherotech, Lake Forest, IL). The absolute number of MPs was calculated using TruCount^TM^ beads (BDB).

### Flow cytometric analysis of adhesion molecule expression on HUVECs

HUVECs were stained with FITC anti-CD105 and APC anti-CD54 (ICAM-1, BDB) for 15 min at RT in dark. Data from at least 10,000 events were acquired and analysed with the use of FACSCalibur flow cytometer and CellQuestPro^TM^ software.

### Analysis of endothelial cell activation markers expression by RT-qPCR

Total mRNA was extracted from HUVECs by Trizol reagent (Thermo Fisher Scientific, Waltham, MA). Then, cDNA was synthesized using Oligo-dT primer and RevertAid Reverse Transcriptase (Thermo Fisher Scientific) from 1 μg of total RNA. Quantification of level of adhesion molecules (ICAM-1, VCAM-1 and E-selectin), coagulation proteins (TF and vWF) and pro-inflammatory cytokines (IL-6 and IL-8) mRNA was analysed by qPCR using a specific TaqMan gene expression assay (Thermo Fisher Scientific, Table S1). The level of target gene expression was normalised against GAPDH expression. The mRNA fold change was calculated using the 2^−ΔΔCt^ method, with the values expressed as fold change relative to the untreated control.

### Tissue factor (TF) activity analysis

The supernatants were collected from 6 h treated HUVECs as described above. TF activity was measured using TF human chromogenic activity assay kit (Abcam) according to the manufacturers’ instructions. TF activity (pM) was determined by reference to a standard curve generated using recombinant TF.

### Analysis of pro-inflammatory cytokines by ELISA

The supernatants were collected from untreated and treated HUVECs as described above. IL-6 and IL-8 levels were measured by using ELISA kits (Sigma-Archrich and BDB, respectively) accordingly to the manufacture’s recommendation.

### Monocyte-endothelial cell adhesion assay

Monocytes adhering to endothelial cells were determined by using fluorescent labeled THP-1 cells. Briefly, 80–90% confluence of HUVECs in a 96-well plate were unprimed or primed with LPS for 1 h followed by addition of 1 × 10^6^ or 5 × 10^6^ particles/mL MPs and incubated for 24 h. Then 2′,7′-bis-(2-carboxyethyl)-5-(and-6)-carboxyfluorescein (BCECF)-labeled THP-1 cells were layered over the Hoechst-labeled HUVECs monolayers for 15 min. Non-adherent THP-1 cells were removed by gently washing. The fluorescent signals of BCECF-labeled THP-1 adherent cells were measured using DTX 880 multimode detector fluorescent plate reader (Beckman Coulter, Brea, CA) with 490/535 nm excitation/emission filter sets. The fluorescent cell morphology was also observed by Ti-S fluorescence inverted microscope (Nikon).

### Statistical analysis

Data were analysed using SPSS Version 18.0 (IBM Collaboration, Armonk, NY) and GraphPad PRISM 6.0 (GraphPad Software, Inc., La Jolla, CA). Comparisons between parameters were evaluated with a non-parametric Mann-Whitney U Test. The correlation coefficient was calculated with Spearman’s Rho (r_s_). The threshold for statistical significance for all comparisons was *P* < 0.05.

## Electronic supplementary material

Supplementary information

## References

[1] Eldor A, Rachmilewitz EA (2002). The hypercoagulable state in thalassemia. Blood.

[2] Taher A (2006). Prevalence of thromboembolic events among 8,860 patients with thalassaemia major and intermedia in the Mediterranean area and Iran. Thromb Haemost.

[3] Borgna Pignatti C (1998). Thromboembolic events in beta thalassemia major: an Italian multicenter study. Acta Haematol.

[4] Cappellini MD (2000). Venous thromboembolism and hypercoagulability in splenectomized patients with thalassaemia intermedia. Br J Haematol.

[5] Moratelli S (1998). Thrombotic risk in thalassemic patients. J Pediatr Endocrinol Metab.

[6] Pazgal I, Inbar E, Cohen M, Shpilberg O, Stark P (2016). High incidence of silent cerebral infarcts in adult patients with beta thalassemia major. Thrombosis research.

[7] Taher AT (2010). Asymptomatic brain magnetic resonance imaging abnormalities in splenectomized adults with thalassemia intermedia. J Thromb Haemost.

[8] Aggeli C (2005). Endothelial dysfunction and inflammatory process in transfusion-dependent patients with beta-thalassemia major. Int J Cardiol.

[9] Butthep P (2002). Increased circulating activated endothelial cells, vascular endothelial growth factor, and tumor necrosis factor in thalassemia. Am J Hematol.

[10] Garnier Y (2017). Differences of microparticle patterns between sickle cell anemia and hemoglobin SC patients. PLoS One.

[11] Lannan KL, Phipps RP, White RJ (2014). Thrombosis, platelets, microparticles and PAH: more than a clot. Drug Discov Today.

[12] Tramontano AF (2010). Circulating endothelial microparticles in diabetes mellitus. Mediators of inflammation.

[13] Chaichompoo P (2012). Characterizations and proteome analysis of platelet-free plasma-derived microparticles in beta-thalassemia/hemoglobin E patients. J Proteomics.

[14] Pattanapanyasat K (2007). Activated platelet-derived microparticles in thalassaemia. Br J Haematol.

[15] Klaihmon P (2017). Microparticles from splenectomized beta-thalassemia/HbE patients play roles on procoagulant activities with thrombotic potential. Ann Hematol.

[16] Atichartakarn V, Chuncharunee S, Chandanamattha P, Likittanasombat K, Aryurachai K (2004). Correction of hypercoagulability and amelioration of pulmonary arterial hypertension by chronic blood transfusion in an asplenic hemoglobin E/beta-thalassemia patient. Blood.

[17] Sirachainan N (2013). Thalassemia and the hypercoagulable state. Thrombosis research.

[18] Tsimerman G (2011). Involvement of microparticles in diabetic vascular complications. Thromb Haemost.

[19] Rautou PE (2011). Microparticles from human atherosclerotic plaques promote endothelial ICAM-1-dependent monocyte adhesion and transendothelial migration. Circ Res.

[20] Camus SM (2015). Circulating cell membrane microparticles transfer heme to endothelial cells and trigger vasoocclusions in sickle cell disease. Blood.

[21] Angchaisuksiri P (2007). Hemostatic and thrombotic markers in patients with hemoglobin E/beta-thalassemia disease. Am J Hematol.

[22] Habib A (2008). Elevated levels of circulating procoagulant microparticles in patients with beta-thalassemia intermedia. Haematologica.

[23] Tantawy AA, Adly AA, Ismail EA, Habeeb NM (2013). Flow cytometric assessment of circulating platelet and erythrocytes microparticles in young thalassemia major patients: relation to pulmonary hypertension and aortic wall stiffness. Eur J Haematol.

[24] Pluskota E (2008). Expression, activation, and function of integrin alphaMbeta2 (Mac-1) on neutrophil-derived microparticles. Blood.

[25] Diehl P (2012). Microparticles: major transport vehicles for distinct microRNAs in circulation. Cardiovasc Res.

[26] Agouti I (2015). Platelet and not erythrocyte microparticles are procoagulant in transfused thalassaemia major patients. Br J Haematol.

[27] Horstman LL, Ahn YS (1999). Platelet microparticles: a wide-angle perspective. Critical reviews in oncology/hematology.

[28] Morel O, Morel N, Freyssinet JM, Toti F (2008). Platelet microparticles and vascular cells interactions: a checkpoint between the haemostatic and thrombotic responses. Platelets.

[29] Tan KT, Tayebjee MH, Lynd C, Blann AD, Lip GY (2005). Platelet microparticles and soluble P selectin in peripheral artery disease: relationship to extent of disease and platelet activation markers. Annals of medicine.

[30] Merten M, Pakala R, Thiagarajan P, Benedict CR (1999). Platelet microparticles promote platelet interaction with subendothelial matrix in a glycoprotein IIb/IIIa-dependent mechanism. Circulation.

[31] Barry OP, Pratico D, Savani RC, FitzGerald GA (1998). Modulation of monocyte-endothelial cell interactions by platelet microparticles. The Journal of clinical investigation.

