# Supplementary material for: Microparticles from β-thalassaemia/HbE patients induce endothelial cell dysfunction
Source: Sci Rep. 2018 Aug 29;8:13033. doi: 10.1038/s41598-018-31386-6 (PMC6115342; doi:10.1038/s41598-018-31386-6)
Supplement: Supplementary file 1 — Supplementary information [file 41598_2018_31386_MOESM1_ESM.pdf]

Supplement information

Microparticles from β-thalassaemia/HbE patients induce endothelial cell dysfunction

Wasinee Kheansaard[1,2], Kunwadee Phongpao[1,2], Kittiphong Paiboonsukwong[1], Kovit Pattanapanyasat[3], Pornthip Chaichompoo[4], Saovaros Svasti[1,5,*]

Table S1. TaqMan gene expression assay for RT-qPCR analysis

Gene	Assay ID, Applied Biosystem
Tissue factor	Hs01076029_m1
von Willebrand factor	Hs01109446_m1
Interleukin-6	Hs00174131_m1
Interleukin-8	Hs00174103_m1
Intracellular adhesion molecule-1	Hs00164932_m1
Vascular cell adhesion molecule-1	Hs01003372_m1
E-selectin	Hs00174057_m1

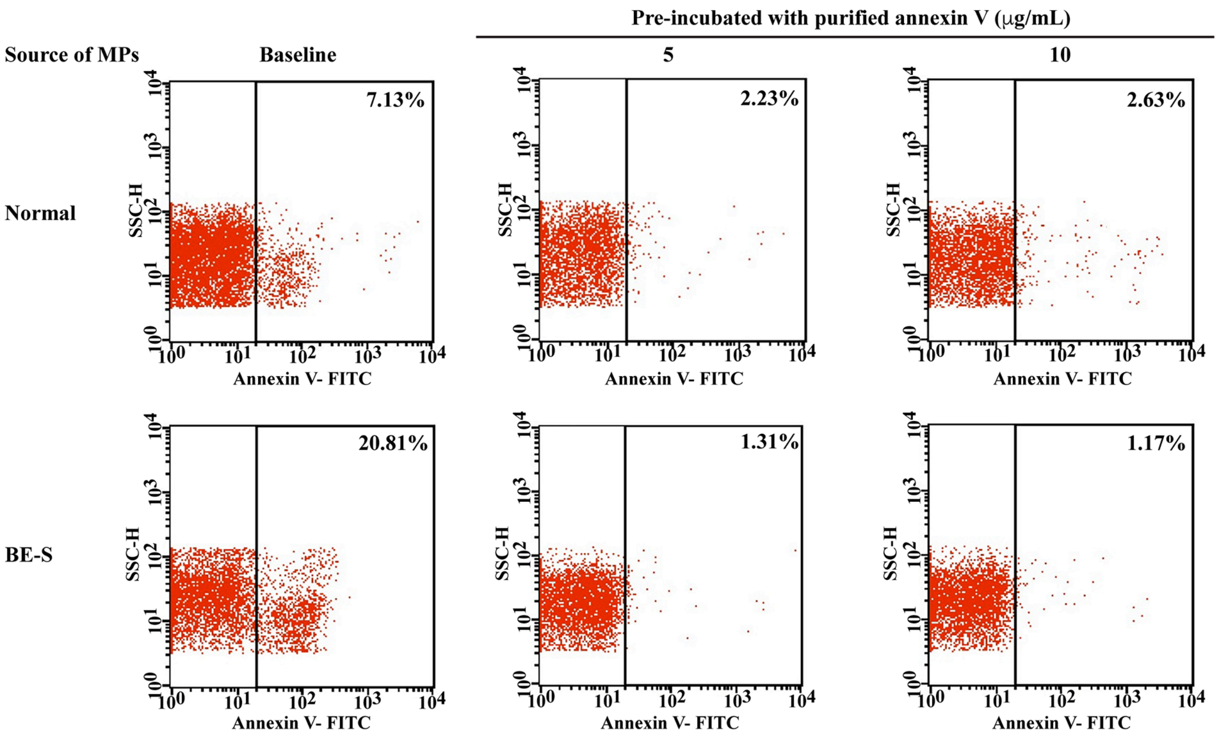

Figure S1. Blocking of phosphatidylserine exposed on microparticles. Isolated MPs from normal subjects and splenectomised β-thalassaemia/HbE patients (BE-S) were incubated with absent or present annexin V at final concentration 5 and 10 µg/mL. MPs were stained with FITC conjugated annexin V and the unblocked PS on MPs were detected by flow cytometry.

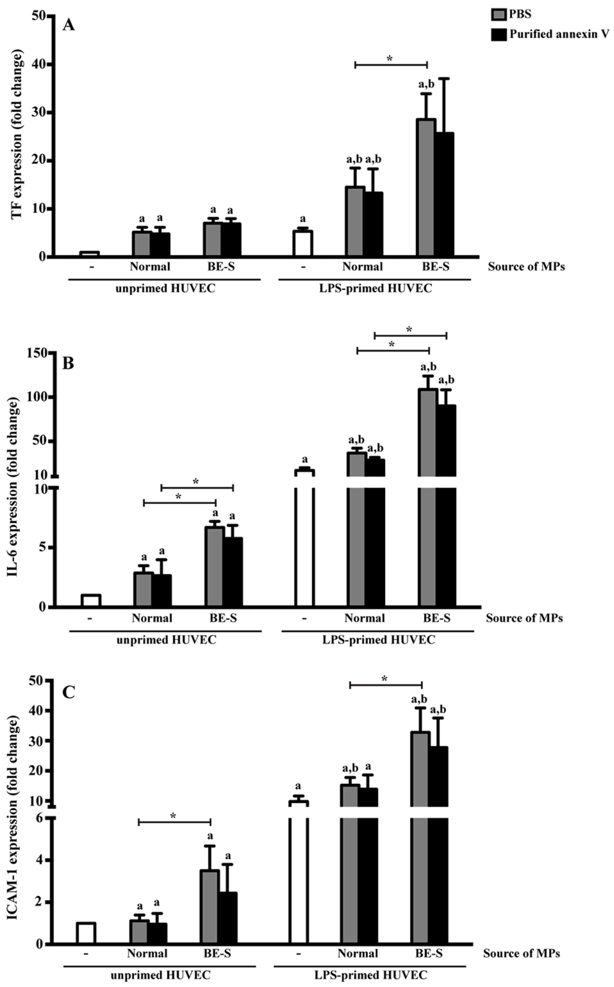

Figure S2. Endothelial dysfunction not caused by phosphatidylserine on MPs. Isolated MPs and annexin V-treated MPs at 5×10^6 particles/mL obtained from 5 normal subjects and 8 splenectomised β-thalassaemia/HbE patients (BE-S) were incubated with unprimed- or LPS-primed HUVECs. The mRNA levels of (A) TF, (B) IL-6 and (C) ICAM-1 in HUVECs were examined by RT-qPCR. [a]Statistically significant difference when compared with unprimed HUVECs at $P < 0.05$. [b]Statistically significant difference when compared with LPS-primed HUVECs at $P < 0.05$. [*]Statistically significant difference between groups at $P < 0.05$. Each experiment was duplicated.

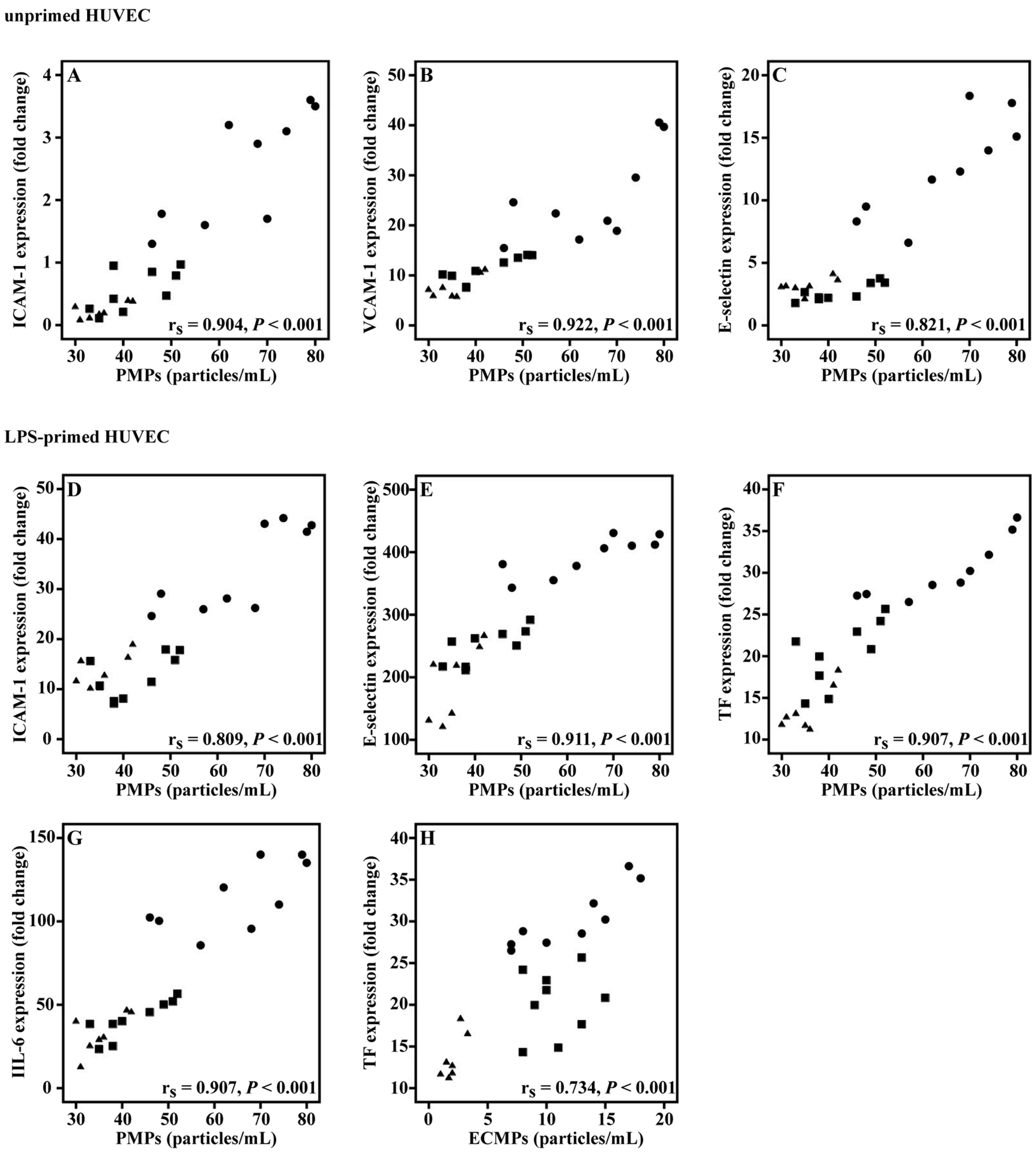

Figure S3. Correlation between MPs origin and endothelial cell activation marker. Correlation between MPs origin and adhesion molecules or cytokine expression in HUVECs by using Spearman's coefficient. Normal subjects; ▲, non-splenectomised β-thalassaemia/HbE patients; ■ and splenectomised β-thalassaemia/HbE patients; ●.
